# Predictive value of dynamic changes in RvD1, sST2, and PIV levels for the prognosis of STEMI patients after PCI: a prospective study

**DOI:** 10.3389/fcvm.2025.1705808

**Published:** 2025-11-14

**Authors:** Yiwei Xu, Li Li, Zejun Lin, Yaning Hu, Yang Liu, Tong Zhou

**Affiliations:** 1 Department of Emergency, The Third Affiliated Hospital of Inner Mongolia Medical University, Inner Mongolia Baogang Hospital, Baotou, China; 2 Department of Cardiovascular Medicine, The Third Affiliated Hospital of Inner Mongolia Medical University, Inner Mongolia Baogang Hospital, Baotou, China; 3 The First Clinical Medical School of Guangzhou University of Chinese Medicine, Guangzhou, China; 4 Department of General Practice, The Third Clinical Medical College of Inner Mongolia Medical University, Inner Mongolia Baogang Hospital, Baotou, China

**Keywords:** ST-segment elevation myocardial infarction, resolvin D1, soluble suppression of tumorigenicity 2, pan-immune-inflammatory value, major adverse cardiovascular events

## Abstract

**Introduction:**

ST-segment elevation myocardial infarction (STEMI) is one of the major subtypes of acute coronary syndrome, with rapid progression and a high risk of death and disability. Although some patients undergo timely percutaneous coronary intervention (PCI), they remain at high risk of major adverse cardiovascular events (MACE) after the procedure. In our study, we analyzed the dynamic levels of resolvin D1 (RvD1), soluble suppression of tumorigenicity 2 (sST2) and the pan-immune-inflammation value (PIV) in STEMI patients within 24 h of disease onset and their prognostic value.

**Methods:**

This prospective cohort study enrolled 184 consecutive STEMI patients between December 2023 and December 2024. Serum RvD1 and sST2 levels were measured by enzyme-linked immunosorbent assay at the time of admission (0 h), 6 h post-onset (6 h), and 24 h post-onset (24 h). PIV was derived from routine laboratory data. All patients were followed for 6 months for the occurrence of MACE.

**Results:**

Dynamic levels of RvD1, sST2, and PIV differed significantly between patients with and without MACE. Multiple logistic regression analysis confirmed that the sST2 level at admission (OR = 1.056, 95% CI: 1.027–1.086), RvD1 level (OR = 0.234, 95% CI: 0.122–0.449) and LnPIV level (OR = 5.139, 95% CI: 1.743–15.154) at 24 h after onset were strong independent risk factors for MACE (all *P* < 0.001). With the inclusion of the Global Registry of Acute Coronary Events Study (GRACE) score in the risk prediction model, the area under the curve (AUC) increased to 0.826 (95% CI: 0.758–0.890), 0.852 (95% CI: 0.786–0.932), and 0.834 (95% CI: 0.750–0.897).

**Conclusion:**

Early dynamic changes in RvD1, sST2, and PIV are significantly associated with prognosis in STEMI patients. These biomarkers, particularly sST2 level at admission, RvD1 and LnPIV levels at 24 h after onset, serve as independent predictors for MACE and could improve risk stratification.

## 1 Introduction

ST-segment elevation myocardial infarction (STEMI), a significant subtype of acute coronary syndrome, is associated with high morbidity and mortality and necessitates urgent revascularization, most commonly through percutaneous coronary intervention (PCI) (1, 2). Despite advances in PCI technology, a considerable proportion of STEMI patients continue to experience major adverse cardiovascular events (MACE) following the procedure (3, 4).

**Figure 1:**
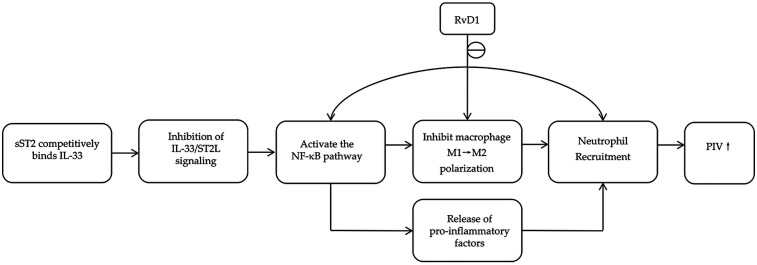
Common pathways involving RvD1, sST2, and PIV.

Inflammation plays a critical role in the pathogenesis and progression of STEMI, leading to myocardial injury, maladaptive cardiac remodeling, and MACE (5). Among the key regulators of inflammatory and fibrotic pathways, the proinflammatory factor soluble suppression of tumorigenicity 2 (sST2), the anti-inflammatory factor resolvin D1 (RvD1), and the pan-immune-inflammation value (PIV) have attracted considerable attention in recent years. As illustrated in Figure 1, the pro-inflammatory mediator sST2 and the pro-resolving mediator RvD1 converge on shared inflammatory pathways, such as the regulation of NF-κB signaling and macrophage polarization. The PIV collectively represents the overall inflammatory state resulting from their interplay (6, 7). All three markers have been established as important biomarkers for prognostic assessment in patients with STEMI (8–10).

Notably, previous investigations have largely been based on single-time-point measurements, thereby constraining a comprehensive assessment of their dynamic temporal profiles after acute myocardial infarction. Dynamic monitoring provides a more comprehensive capture of pathophysiological processes. By revealing the temporal dynamics of biomarkers, it significantly enhances the precision of risk stratification, thereby identifying covert high-risk patients who would be underestimated by a single-time point assessment. Moreover, current evidence primarily stems from nonemergent or non-STEMI patient groups, raising doubts about the applicability of these findings to patients with acute STEMI, these limitations hinder the translational potential of these biomarkers for risk stratification and tailored treatment strategies in STEMI patients (11–13).

This study aims to dynamically monitor early-phase serum levels of RvD1, sST2, and PIV in STEMI patients to characterize their temporal profiles within the acute stage. By evaluating these biomarkers from both pro-inflammatory and anti-inflammatory perspectives, we seek to systematically assess their respective values in determining disease severity and predicting clinical outcomes. Our findings are expected to provide a theoretical foundation for optimizing risk stratification and prognostic evaluation in STEMI patients.

## 2 Materials and methods

### 2.1 Study population

This study consecutively enrolled 184 patients with STEMI who presented to the Emergency Department of the Third Affiliated Hospital of Inner Mongolia Medical University and subsequently underwent PCI in the Department of Cardiology from December 2023 to December 2024.

The inclusion criteria were as follows: (1) age ≥ 18 years; (2) diagnosis of STEMI confirmed according to the criteria established by the American College of Cardiology (ACC) and the European Society of Cardiology (ESC); and (3) presentation to the emergency department of our institution within 3 h of symptom onset, with coronary angiography and PCI performed by an experienced cardiologist within 6 h after symptom onset.

The exclusion criteria were as follows: (1) delay of more than 3 h between chest pain onset and hospital admission; (2) presence of cardiac diseases other than STEMI; (3) death during hospitalization; (4) incomplete PCI procedure; (5) incomplete baseline data; (6) history of previous myocardial infarction or primary cardiomyopathy; (7) severe hepatic or renal dysfunction or significant electrolyte disturbances; and (8) active infectious diseases.

**Figure 2:**
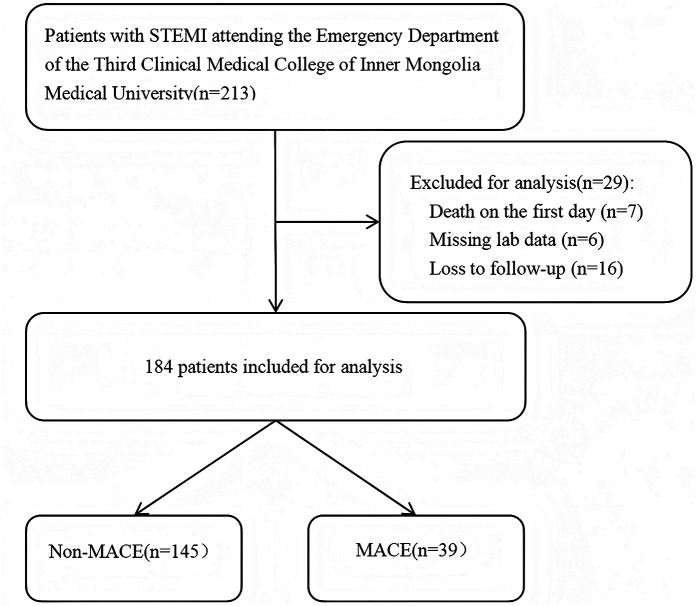
The flowchart of patient selection.

This study was approved by the Ethics Committee of the Third Affiliated Hospital of Inner Mongolia Medical University (Approval No. 2022-MER-041) and was conducted in accordance with the principles of the Declaration of Helsinki. Informed consent was obtained from all participants. The patient selection flowchart is presented in Figure 2.

### 2.2 Data collection

The research team collected all demographic information, medical history, vital signs, and initial laboratory data immediately upon patient admission. Venous blood samples (3 mL) were drawn from the anterior elbow vein and sampled at three time points: at admission (labeled 0 h), 6 h after symptom onset (post-PCI was required), and 24 h after symptom onset for the three biomarkers. The serum levels of sST2 and RvD1 were measured via enzyme-linked immunosorbent assay, while the PIV was calculated on the basis of complete blood count results provided by the hospital's clinical laboratory, using the formula: neutrophil count (10^9^/L) × platelet count (10^9^/L) × monocyte count (10^9^/L)/lymphocyte count (10^9^/L). To satisfy the homoscedasticity assumption of linear models and reduce distributional skewness, the PIV underwent natural logarithmic transformation (LnPIV). All treatment protocols were documented by cardiologists. A data monitoring committee regularly reviewed the accuracy of all collected data, and the overall missing data rate was less than 5%.

### 2.3 Follow-up and endpoints

Follow-up data were collected by cardiologists through telephone interviews, outpatient visits, or medical record reviews. The primary endpoint of this study was the incidence of MACE during the follow-up period, which included malignant arrhythmias, severe heart failure, recurrent nonfatal myocardial infarction, and all-cause mortality (14). All potential endpoint events were reviewed and confirmed by an independent Clinical Events Committee, blinded to the biomarker data, whose members were not otherwise involved in the study.

### 2.4 Statistical analysis

All the statistical analyses were performed via SPSS (version 27.0) and R software (version 4.5.1), with statistical significance defined as *P* < 0.05. Continuous variables with a normal distribution are presented as mean ± standard deviation, while those with a non-normal distribution are shown as median and interquartile range. Differences between the MACE and non-MACE groups were assessed using independent samples *t*-tests for normally distributed variables and the Mann–Whitney *U* test for non-normally distributed variables. Categorical variables are summarized as counts (%) and compared using the chi-square test. Each biomarker was dichotomized into high-level and low-level groups on the basis of its median value. Kaplan–Meier (K–M) survival curves were plotted, and between-group differences in survival were assessed via the log-rank test. The predictive value of each biomarker for MACE was evaluated via receiver operating characteristic (ROC) curve analysis. Restricted cubic splines (RCSs) were applied for smooth fitting to explore the continuous associations between biomarker levels and the probability of MACE occurrence. Threshold effect analysis was conducted to determine the optimal cutoff value of each biomarker for predicting MACE.

## 3 Results

### 3.1 Patient characteristics and baseline data

This study included 184 patients, with 145 assigned to the non-MACE group (mean age 59.16 ± 12.81 years) and 39 to the MACE group (mean age 65.26 ± 14.54 years). Comparative analysis of baseline characteristics revealed statistically significant differences in Age; RBC; Urea level; GRACE score; RvD1 and sST2 and LnPIV levels at each measured time point (all *P* < 0.05). No other parameters were significantly different between the two groups (Table 1). Notably, all enrolled patients were admitted within 3 h after symptom onset; as a result, the majority did not exhibit elevated troponin levels at the initial assessment, which may explain the lack of a significant difference in troponin values between the groups (*P* = 0.490).

**Table 1:** Baseline clinical characteristics of the MACE and non-MACE groups.

Variables	Non-MACE (145)	MACE (39)	*P*
Age (years)	59.16 ± 12.81	65.26 ± 14.54	**0** **.** **011**
Males, *n* (%)	116 (80.00)	33 (84.62)	0.514
Heart rate (beats/min)	75.00 (65.00–84.00)	74.00 (68.00–79.00)	0.995
SPB (mmHg)	128.95 ± 24.56	129.51 ± 26.47	0.996
DPB (mmHg)	80.90 ± 14.85	81.18 ± 15.84	0.867
Current smoker, *n* (%)	73 (50.35)	16 (41.03)	0.301
Current drink, *n* (%)	39 (26.90)	9 (23.08)	0.630
Hypotension history, *n* (%)	73 (50.35)	24 (61.54)	0.214
Diabetes history, *n* (%)	37 (25.52)	12 (30.77)	0.510
Laboratory tests			
RBC (×10^9^/L)	4.95 (4.66–5.34)	4.79 (4.39–5.09)	**0** **.** **037**
WBC (×10^9^/L)	8.33 (6.79–10.73)	9.15 (7.08–12.21)	0.223
TC (mmol/L)	4.72 (3.80–5.23)	4.32 (3.73–5.04)	0.283
HDL-C (mmol/L)	1.01 (0.85–1.26)	1.12 (0.92–1.22)	0.745
LDL-C (mmol/L)	3.05 (2.17–3.72)	2.77 (2.33–3.48)	0.402
Glucose (mmol/L)	6.37 (5.30–8.76)	7.28 (5.56–9.00)	0.292
SCr (μmol/L)	77.100 (67.70–92.10)	80.30 (72.80–91.40)	0.282
Urea (mmol/L)	5.90 (4.70–7.10)	6.30 (5.50–7.90)	**0** **.** **023**
ALT (U/L)	25.00 (18.00–35.00)	27.00 (16.00–43.00)	0.775
D-Dimer (μg/mL)	0.34 (0.29–0.44)	0.35 (0.30–0.55)	0.428
cTn (ng/mL)	0.11 (0.01–1.90)	0.21 (0.01–7.23)	0.490
NT-proBNP (pg/mL)	360.00 (80.00–984.00)	567.00 (109.00–1,985.00)	0.098
Medications after discharge			
ACEI/ARB, *n* (%)	98 (67.59)	25 (64.10)	0.682
B-blockers, *n* (%)	120 (82.76)	37 (94.87)	0.058
CCB, *n* (%)	13 (8.97)	2 (5.13)	0.437
Nitrose, *n* (%)	49 (33.79)	17 (43.59)	0.257
Nicorandil, *n* (%)	32 (22.07)	13 (33.33)	0.146
Trimetazidine, *n* (%)	14 (9.655)	5 (12.821)	0.564
Statins, *n* (%)	144 (99.31)	39 (100.00)	0.603
Loop diuretic, *n* (%)	65 (44.83)	21 (53.85)	0.316
Culprit vessels, *n* (%)			
LAD	128 (88.28)	34 (87.18)	0.851
LCX	91 (62.76)	28 (71.80)	0.295
RCA	80 (55.17)	22 (56.41)	0.890
Number of diseased coronary vessels, *n* (%)			
1	45 (31.03)	10 (25.64)	0.514
2	43 (29.66)	13 (33.33)	0.658
3	56 (38.62)	16 (41.03)	0.785
Study parameters			
GRACE	116.22 ± 29.32	138.95 ± 29.69	**<0** **.** **001**
RvD1 0 h	6.89 (5.67–8.43)	5.63 (5.14–6.69)	**0** **.** **002**
RvD1 6 h	5.98 (4.80–7.12)	4.95 (4.11–6.18)	**0** **.** **007**
RvD1 24 h	7.32 (6.07–8.88)	4.82 (4.47–5.69)	**<0** **.** **001**
sST2 0 h	17.70 (10.45–26.38)	38.66 (19.58–59.05)	**<0** **.** **001**
sST2 6 h	40.79 (21.10–67.23)	60.58 (38.26–80.14)	**0** **.** **011**
sST2 24 h	29.08 (14.02–51.80)	52.47 (18.34–71.39)	**0** **.** **035**
LnPIV 0 h	5.58 (5.17–6.27)	6.39 (5.97–6.67)	**<0** **.** **001**
LnPIV 6 h	6.20 (5.55–6.69)	6.70 (6.42–7.10)	**<0** **.** **001**
LnPIV 24 h	5.95 (5.49–6.39)	6.82 (6.09–7.31)	**<0** **.** **001**

SBP, systolic blood pressure; DBP, diastolic blood pressure; RBC, red blood cell count; WBC, white blood cell count; TC, total cholesterol; HDL-C, high-density lipoprotein cholesterol; LDL-C, low-density lipoprotein cholesterol; SCr, serum creatinine; ALT, alanine aminotransferase; NT-proBNP, N-terminal pro-B-type natriuretic peptide; ACEI, angiotensin-converting enzyme inhibitor; ARB, angiotensin II receptor blocker; CCB, calcium channel blocker; LAD, left anterior descending artery; LCX, left circumflex artery; RCA, right coronary artery; 0 h, at admission; 6 h, 6 h after symptom onset (post-PCI); 24 h, 24 h after symptom onset.

The bold values indicate statistical significance (*P* < 0.05).

### 3.2 Associations of dynamic changes in sST2, RvD1 and LnPIV within 24 h of onset with MACE in STEMI patients

**Figure 3:**
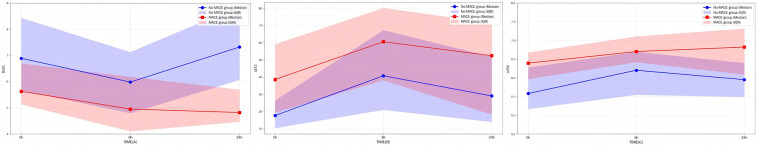
**(A–C)** The dynamic trends of three indicators within 24 h, with shaded areas indicating 95% confidence intervals.

During the first 24 h after STEMI onset, distinct dynamic patterns of sST2, RvD1, and LnPIV were observed between the two groups. Throughout this period, the MACE group consistently presented significantly higher levels of sST2 and LnPIV, along with lower levels of RvD1, than did the non-MACE group. The level of sST2 initially increased but then subsequently decreased in both groups. In contrast, RvD1 initially decreased but then rebounded in the non-MACE group, whereas it continued to decrease persistently in the MACE group. Similarly, LnPIV demonstrated an early increase followed by a decrease in the non-MACE group, whereas it exhibited a sustained upward trend in the MACE group (Figure 3).

### 3.3 Independent predictors of MACE

After several laboratory indicators were excluded because of collinearity issues, the remaining variables were included in the univariate and multivariate logistic regression analyses (Table 2). Univariate analysis revealed that Age, TG, NT-proBNP, RBC, GRACE, RvD1, sST2, and LnPIV at all measured time points were significantly associated with MACE (all *P* < 0.05). Multivariate logistic regression further confirmed that TG, GRACE, RvD1 6 h, RvD1 24 h, sST2 0 h, and LnPIV 24 h were independent risk factors for MACE in STEMI patients (all *P* < 0.05). Notably, GRACE, RvD1 24 h, sST2 0 h, and LnPIV 24 h were identified as strong independent risk factors (all *P* < 0.01).

**Table 2:** Univariate and multivariate logistic regression analyses of factors associated with STEMI.

Variable	Univariate logistic regression			Multivariate logistic regression		
	OR	95% CI	*P* value	OR	95% CI	*P* value
Age	1.036	1.007–1.065	0.014			
TG	0.475	0.259–0.783	0.008	0.297	0.088–0.833	0.032
NT-proBNP	1.000	1.000–1.001	0.002			
RBC	0.578	0.346–0.966	0.036			
GRACE	1.027	1.014–1.041	<0.001	1.057	1.019–1.107	0.007
RvD1 0 h	0.76	0.614–0.922	0.008			
RvD1 6 h	0.749	0.592–0.929	0.012	3.770	1.330–13.600	0.023
RvD1 24 h	0.396	0.275–0.538	<0.001	0.164	0.057–0.364	<0.001
sST2 0 h	1.045	1.026–1.065	<0.001	1.079	1.041–1.131	<0.001
sST2 6 h	1.017	1.004–1.029	0.010			
sST2 24 h	1.016	1.003–1.029	0.017			
LnPIV 0 h	2.458	1.556–4.026	<0.001			
LnPIV 6 h	2.657	1.597–4.653	<0.001			
LnPIV 24 h	5.395	2.986–10.813	<0.001	9.717	2.322–58.08	0.005

### 3.4 Subgroup analysis

Subgroup analyses were performed on the basis of sex, age, and history of hypertension or diabetes to evaluate the significant differences in the three strong predictors—RvD1 24 h, sST2 0 h, and LnPIV 24 h—across different patient populations (Table 3). The results indicated that sST2 0 h had a significantly greater predictive ability in male patients [1.046 (1.025, 1.068), *P* < 0.0001] than in female patients [1.032 (0.979, 1.087), *P* = 0.2472] and was less applicable in patients with a history of diabetes [1.006 (0.977, 1.034), *P* = 0.7034]. No statistically significant differences were observed for the other indicators across subgroups (all *P* < 0.05).

**Table 3:** Associations between sST2 0 h, RvD1 24 h, and LnPIV 24 h and MACE across different subgroups.

Subgroup	sST2 0 h	RvD1 24 h	LnPIV 24 h
Stratified by gender, OR (95% CI), *P* value			
Male	1.046 (1.025, 1.068) <0.0001	0.395 (0.275, 0.568) <0.0001	5.092 (2.556, 10.146) <0.0001
Female	1.032 (0.979, 1.087) 0.2472	0.402 (0.180, 0.898) 0.0263	7.214 (1.290, 40.359) 0.0245
Stratified by age, OR (95% CI), P value			
<60	1.073 (1.036, 1.111) <0.0001	0.542 (0.368, 0.799) 0.0020	4.770 (1.691, 13.455) 0.0031
≥60	1.033 (1.008, 1.059) 0.0097	0.249 (0.133, 0.464) <0.0001	5.658 (2.465, 12.991) <0.0001
History of diabetes, OR (95% CI), P value			
Yes	1.006 (0.977, 1.034) 0.7034	0.189 (0.066, 0.543) 0.0020	6.027 (1.442, 25.183) 0.0138
No	1.076 (1.046, 1.108) <0.0001	0.447 (0.315, 0.634) <0.0001	5.338 (2.605, 10.941) <0.0001
History of hypertension, OR (95% CI), P value			
Yes	1.029 (1.006, 1.053) 0.0138	0.375 (0.236, 0.597) <0.0001	5.168 (2.256, 11.838) 0.0001
No	1.070 (1.034, 1.108) 0.0001	0.387 (0.226, 0.660) 0.0005	5.512 (2.049, 14.829) 0.0007

### 3.5 Restricted cubic spline (RCS)

**Figure 4:**
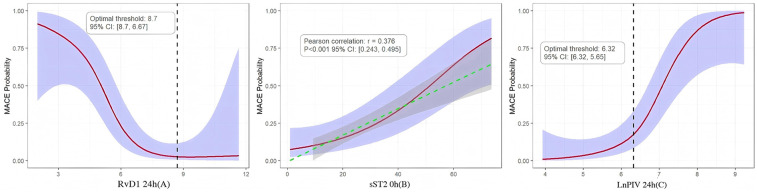
**(A–C)** RCS of the relationships between RvD1 24 h, sST2 0 h, LnPIV 24 h and MACE probability, overlaid with 95% confidence intervals (purple shaded areas).

Analysis results indicate that RvD1 24 h exhibit a negative correlation with MACE, demonstrating a clear threshold effect. The optimal risk threshold is 8.7 ng/mL. When RvD1 levels fell below this threshold, the probability of MACE occurrence increased sharply, this not only established for the first time a clinically actionable RvD1 risk cutoff value but also confirmed that insufficient levels of pro-inflammatory resolution mediators represent a key mechanism underlying poor prognosis. Concurrently, sST2 0 h showed a significant linear positive correlation with MACE (Pearson *r* = 0.376, 95% CI: 0.243–0.495). Higher sST2 levels consistently correlated with increased MACE, indicating that early myocardial stress and fibrosis signals represent persistent risk factors driving adverse outcomes. Furthermore, LnPIV 24 h showed a significant positive correlation with MACE, with an optimal risk threshold of 6.32. Beyond this threshold, MACE risk exhibited a steep upward trend, indicating that failure to effectively control systemic inflammation within the first day post-onset directly leads to a significant increase in the risk of clinical events. This threshold provides critical evidence for identifying patients at extremely high risk (Figure 4).

### 3.6 The ability of sST2 0 h, RvD1 24 h, and LnPIV 24 h alone and in combination with the GRACE score to predict MACE

**Figure 5:**
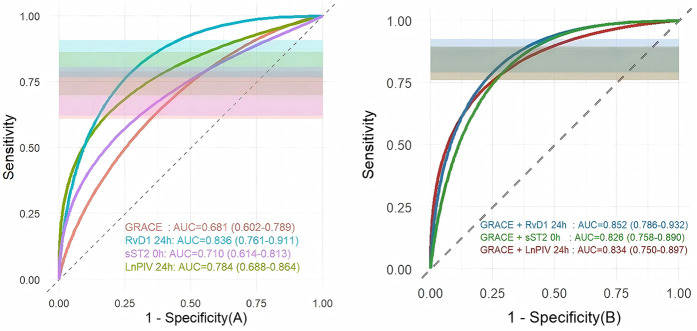
Comparison of ROC curve analysis, overlaid with 95% confidence intervals (purple shaded areas): **(A)** comparison of ROC curves between three indicators and the GRACE score; **(B)** comparison of ROC curves between three indicators combined with the GRACE score.

ROC curve analysis demonstrated that both the individual biomarkers and their combinations with the GRACE score exhibited significant predictive value for MACE (Figure 5). Among the individual predictors, RvD1 24 h had the highest discriminatory ability, with an AUC of 0.836 (95% CI: 0.761–0.911), followed by LnPIV 24 h (AUC = 0.784, 95% CI: 0.688–0.864) and sST2 0 h (AUC = 0.710, 95% CI: 0.614–0.813), all of which outperformed the GRACE score alone (AUC = 0.681, 95% CI: 0.602–0.789). Among all the combined models, the combination of the GRACE score with RvD1 24 h achieved the highest predictive performance, with an AUC of 0.852 (95% CI: 0.786–0.932). This suggests it may serve as a crucial strategy for clinical risk prediction and prognosis assessment in STEMI patients.

### 3.7 Survival analysis

**Figure 6:**
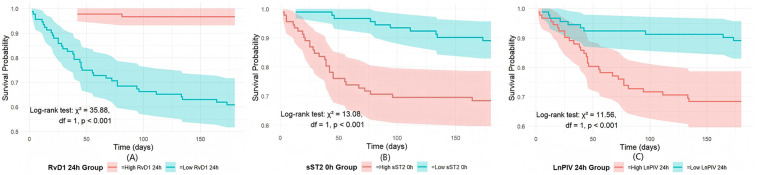
Comparison of the three indicators via K‒M survival analysis: **(A)** K–M survival curves by RvD1 24 h level; **(B)** K–M survival curves by sST2 0 h level; **(C)** K–M survival curves by LnPIV 24 h level.

This study divided each indicator into low-level and high-level groups based on the median and performed K–M survival curve analysis (Figure 6). The results showed a significant difference between the two groups. Patients with high RvD1 24 h levels had significantly better survival than those with low RvD1 24 h levels (log-rank *χ*^2^ = 35.88, df = 1, *P* < 0.001), confirmed that lower RvD1 24 h levels are a strong predictor of poor long-term prognosis. Conversely, elevated sST2 0 h levels were associated with poorer survival rates (log-rank *χ*^2^ = 13.08, df = 1, *P* < 0.001), underscoring the critical role of acute myocardial stress responses in prognostic assessment. Similarly, the significant survival difference between the LnPIV 24 h groups (Log-rank *χ*^2^ = 11.56, *P* < 0.001) further underscores that persistent high-grade inflammation is strongly associated with worsening long-term clinical outcomes.

## 4 Discussion

In this study, we revealed the value of RvD1, sST2 and PIV in predicting MACE by dynamically monitoring the changes in their levels within 24 h after the onset of STEMI. It was found that sST2 peaked rapidly early, RvD1 showed a depletion-recovery trajectory, and PIV reflected a net inflammatory state. These dynamic changes were more pronounced in the MACE group, suggesting the limitations of previous single time-point assessments and emphasizing the importance of repeated sampling during the acute phase. RvD1 24 h <8.7 and LnPIV 24 h >6.32 were also calculated to be the best thresholds for predicting MACE, suggesting that early control of inflammation can help improve prognosis and provide a basis for targeted therapy. For example, those with low RvD1 can be supplemented with pro-repair mediators in a timely manner, while those with high sST2 or LnPIV need timely and intensive anti-inflammatory and antifibrotic therapy.

These biomarkers are able to capture the dynamically evolving inflammatory and repair processes, thus compensating for the major shortcomings of traditional scoring systems such as TIMI, HEART and GRACE scores—they are mostly based on static clinical indicators, which are difficult to reflect pathophysiological changes in real time and the scoring process is more cumbersome (15). The joint model constructed by the GRACE score in the study showed that the joint prediction effect was significantly better than that of a single GRACE score, which further confirmed that combining dynamic biomarkers with traditional scoring not only improves the accuracy of risk stratification, but also opens up a new direction for the clinical translation of individualized interventions (16, 17).

As a key regulator in the resolution of inflammation, the lipid mediator RvD1 has garnered significant attention for its sources and functions. Existing research indicates that RvD1 primarily promotes inflammation resolution by enhancing macrophage phagocytic capacity and modulating the expression of chemokines and anti-inflammatory genes (18–20). Chen's study showed that elevated levels of RvD1 were strongly associated with coronary plaque instability and exhibited significant level changes during the initial phase of STEMI. This study revealed an initial decrease followed by a rebound in RvD1 levels in the early stage of acute STEMI. The underlying mechanism may involve rapid consumption of baseline RvD1 due to the abrupt and severe inflammatory response at onset, followed by gradual recovery through compensatory endogenous RvD1 synthesis. However, in severely affected patients, high levels of inflammatory cytokines may suppress this compensatory mechanism, resulting in persistently low RvD1 levels within 24 h after onset. Therefore, the RvD1 level at 24 h post-onset may serve as a more reliable prognostic indicator (21–23).

sST2, a member of the interleukin-1 receptor family, competitively inhibits the IL-33/ST2l pathway by acting as a decoy receptor, thereby exacerbating inflammation, delaying its resolution, and promoting myocardial injury (24–26). Multiple studies have confirmed that elevated sST2 levels in the acute phase of STEMI are closely associated with enhanced inflammatory responses, impaired cardiac repair, and adverse clinical outcomes. Its value in early risk stratification and prognostic evaluation in patients with acute STEMI has been widely validated (27–29). In this study, we found that sST2 was significantly elevated in some patients before serum troponin levels were elevated at the time of admission, suggesting that sST2 may be superior to traditional biomarkers for very early diagnosis and risk assessment of STEMI (30, 31). Furthermore, sST2 levels within 24 h post-STEMI exhibit a characteristic rapid rise and fall. This initial surge likely results from the acute release of sST2 from stressed cardiomyocytes, fibroblasts, and vascular endothelial cells, with peak concentrations indicating more severe vascular injury. Following reperfusion therapy and partial anti-inflammatory factors, as well as its relatively short half-life (about 2–3 h), sST2 levels decline rapidly (32, 33). Subgroup analyses further revealed that the predictive performance of sST2 was modified by sex and diabetes status. The attenuated association observed in female patients may be attributed not only to the limited sample size of women in this acute STEMI cohort but also to the known anti-fibrotic and anti-inflammatory properties of estrogen, which may suppress activity of the sST2/IL-33 pathway (34). In diabetic patients, chronic low-grade inflammation may lead to consistently elevated sST2, reducing its discriminative power for acute events (35).

PIV has been shown to have unique predictive ability for complications such as coronary artery microthrombosis and in-stent restenosis, and has shown significant prognostic value, especially in high-risk diabetic populations (36, 37). Based on existing studies, our study innovatively investigated the dynamic changes of LnPIV in the early stages of acute ST-segment elevation myocardial infarction and its relationship with long-term prognosis, thus effectively reducing the impact of outliers (38). The results demonstrated that substantial release of inflammatory cytokines in the acute phase led to a significant increase in PIV. Persistently elevated PIV levels 24 h after onset are often associated with poorer prognosis. This finding is supported by the results of our threshold effect analysis. Therefore, the LnPIV level 24 h after onset in STEMI patients serves as a significant predictor of MACE, underscoring the importance of controlling the early inflammatory response.

Despite the significant findings of this study, several limitations should be acknowledged. First, the single-center design and relatively limited sample size may introduce selection bias and restrict the generalizability and external validity of the results. Future multicenter, large-scale prospective studies are warranted to validate our findings. Second, owing to budgetary and logistical constraints, biomarker measurements were conducted only at three fixed time points after symptom onset, which may not capture more detailed dynamic changes, such as peak concentrations or fluctuation rates. Subsequent investigations incorporating more frequent early sampling would help clarify the temporal profiles of these biomarkers during the acute phase and their associations with clinical outcomes. Third, our findings may be influenced by survivorship bias because we excluded patients who died during hospitalization. This may have resulted in an underestimation of the true mortality risk and an overestimation of favorable outcomes in our cohort. Furthermore, since there are far more male than female patients with acute STEMI, this limitation prevented us from performing a robust gender-specific assessment. Future studies should aim to recruit gender-balanced populations to explore more targeted gender-specific risk assessment strategies. Despite these limitations, this study provides initial insight into the dynamic changes in and prognostic value of RvD1, sST2, and PIV in the acute phase of STEMI. Building on these results, further mechanistic research and the development of integrated predictive models are encouraged.

## 5 Conclusions

During the first 24 h following STEMI onset, the levels of RvD1, sST2, and PIV exhibit distinct dynamic patterns, with significant differences observed between patients who experienced MACE and those who did not. Among these biomarkers, RvD1 24 h, sST2 0 h, and LnPIV 24 h were identified as independent predictors of MACE. When combined with the GRACE score, they further enhanced the predictive performance. Specifically, sST2 0 h showed a positive linear correlation with MACE risk; RvD1 24 h demonstrated a nonlinear negative correlation with MACE, with levels above 8.7 associated with a significant reduction in MACE incidence; and LnPIV 24 h exhibited a nonlinear positive correlation, with values exceeding 6.32 significantly increasing the risk of MACE. Moreover, significant survival differences were observed between the low-level and high-level groups for each of these indicators.

## Data Availability

The datasets presented in this article are not readily available because they are subject to institutional restrictions and cannot be shared publicly. Requests to access the datasets should be directed to Tong Zhou, joant69@126.com.
